# Insights from the Perspective of Traditional Chinese Medicine to Elucidate Association of Lily Disease and Yin Deficiency and Internal Heat of Depression

**DOI:** 10.1155/2020/8899079

**Published:** 2020-11-25

**Authors:** Bingxian Shang, Hongxiu Zhang, Yanting Lu, Xiaoyu Zhou, Yong Wang, Minghan Ma, Ke Ma

**Affiliations:** ^1^Shandong Co-Innovation Center of Classic TCM Formula, Shandong University of Traditional Chinese Medicine, Jinan 250355, China; ^2^College of Chinese Pharmacy, Beijing University of Chinese Medicine, Beijing 100029, China; ^3^Institute of Virology, Jinan Municipal Center for Disease Control and Prevention, Jinan 250021, China

## Abstract

Lily disease was first recorded in *Synopsis of the Golden Chamber* by Zhang Zhongjing. It is a disease of heart and lung internal heat by Yin deficiency, which belongs to the category of emotion disease in Chinese medicine. In recent years, researchers believe that lily disease and depression syndrome of Yin deficiency and internal heat have many similarities in etiology, pathogenesis, and clinical manifestations. This review summarizes the clinical symptoms, etiology, pathogenesis, and therapeutic medication of lily disease and modern Yin-deficient internal heat depression and discusses the relationship between them. Furthermore, the relationship between coronavirus disease 2019 (COVID-19) and lily disease was discussed from the etiology, pathogenesis, and treatment. It provides new ideas for the treatment of COVID-19 and the treatment of psychological problems after recovery.

## 1. Introduction

Major depression disorder (MDD) is a mood disorder characterized by sentimentality, despair, anhedonia, and sensitivity for social rejection [1]. These symptoms severely debilitated the patient's physical and mental homeostasis and brought a significant reduction in productivity and negative effects on overall health [2]. In terms of treatment and care costs for depression, the World Health Organization (WHO) has predicted it as a leading disease burden worldwide by 2030 [3].

Emerging evidences from human postmortem and animal studies point out depressive disorders may instigate from human body system imbalance affected by genetic and environmental factors [4]. However, the specific pathogenesis underlying the development of depression remains unclear. Currently, psychotherapy and antidepressant medications are the gold-standard treatment for depression in clinical practice [5]. Although antidepressant drugs are convenient treatments for depression, long-term side effects and drug dependence make patients less compliant with them. Hence, it is necessary to find novel therapies which may avert or impede the development of depression to replace conventional Western medicine.

As a critical component of complementary and alternative medicine, traditional Chinese medicine (TCM) is a multicomponent, multitarget, and multipathway therapy, which can achieve its unique therapeutic effect by adjusting the biological network of the human body system [6, 7]. TCM has been used to treat depressive disorders for more than two thousands of years and now has extensive scientific evidences supporting its efficacy [8]. Based on the TCM theory, depression can be divided into positive syndromes and deficiency syndromes, including Qi stagnation, fire stagnation, phlegm stagnation, blood stagnation, heart and spirit loss nourish, deficiency of both heart and spleen, Yin deficiency, and fire excess [9].

Most of Yin deficiency and internal heat of depression is the deficiency syndrome, and its main clinical manifestations are anxiety, depression, tension, suspicion, irritated fever, night sweat and zygomatic red, palpitation and insomnia, red tongue with little moss, and thready and rapid pulse [10].

Lily disease was first seen in *Synopsis of the Golden Chamber* by Zhang Zhongjing. It is a disease of heart and lung internal heat by Yin deficiency, which belongs to the category of emotion disease in Chinese medicine [11]. In recent years, researchers believe that lily disease and depression syndrome of Yin deficiency and internal heat have many similarities in etiology, pathogenesis, and clinical manifestations. This review summarizes the clinical symptoms, etiology, pathogenesis, and therapeutic medication of lily disease and modern Yin-deficient internal heat depression and discusses the relationship between them.

## 2. Lily Disease

Based on the TCM theory, many disease occurrences are related to personal physical and emotional injury [12]. It will cause visceral dysfunction and the imbalance of Yin, Yang, Qi, and blood, when people are in long-term emotional stimulation or mental stimulation beyond the range of physical regulation can bear, leading to the occurrence of diseases [13]. According to the Chinese medicine principle, viscera are closely related to emotional activities. The vital substance of viscera is the material basis of emotional activity, and abnormal emotional activities can damage the vital substance of viscera, resulting in the disorder of Qi and blood, and the imbalance of Yin and Yang [14].

The “lily disease” is initially reported in *Synopsis of the Golden Chamber*: *Lily Disease, Huhuo and Yin Yang Toxin: Pulses, Syndromes and Treatment*. The manifestations of “lily disease” patients are marked by unclear consciousness, fluctuated appetite, frequent silence, restlessness, confused cold and heat body sensation, bitterness in the mouth and dark urine, red tongue body and less tongue coating, and weak and thready pulse. These characteristics are similar to the manifestations of depressive disorders [11]. Based on the TCM theory, “lily disease” results from Yin-Yang imbalance on the whole body, which causes heat and lung disorder. Endogenous heat (heart and lungs) caused by Yin deficiency, resulting in the abnormality of blood vessels and meridians, can be attributed to the pathophysiology of “lily disease” [15].

Emerging evidences from the ancient Chinese medical text suggest that the etiology factor of lily disease is mainly internal and external injury [16]. The internal cause is excessive thinking and emotional failure, so that the spleen, lung, liver, and other visceral functions are damaged, and then the whole body with diseases; the external factor is exogenous heat resulting in body residual heat, which leads to the lack of blood supply and mental confusion [17].

**Figure 1:**
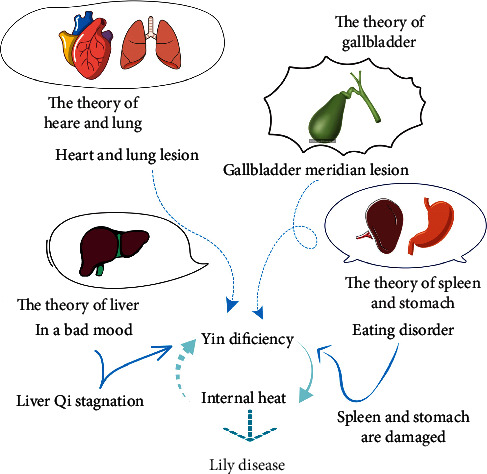
TCM hypothesis mechanism underlying internal and external factors inducing lily disease.

With regard to pathogenesis, there are many TCM hypothesis mechanisms underlying internal and external factors inducing lily disease (Figure 1). The most mainstream view is the theory of heart and lung. Scholars believe that the lung is in charge of all the meridians, the heart governs the blood, and the meridians hold the blood [18]. Thus, lily disease is the disease of the heart and lung. Heart and lung function in the human body is very important, if it is normal, then Qi and blood will be harmonized, and meridians will be nourished; if the heart, lung, Qi, and blood are damaged, the meridians will lose nourishment, and the invasion of exogenous pathogens factors will disturb the stomach, gallbladder, liver, kidney, and other viscera, leading to systemic diseases; as a result, many viscera show symptoms of disease and the position is not fixed [19].

The second view is the theory of gallbladder. For example, Liang et al. believe that “bitterness in the mouth” is a syndrome of Shaoyang gallbladder meridian disease [20]. *Plain questions* said that “all eleven dirty depends on the gallbladder.” Therefore, if the gallbladder has heat, all veins are affected by Yin deficiency. The gallbladder controls the decision, so the deficiency of gallbladder Qi showed the symptoms of trance. The third view is the theory of liver. Some scholars believe that the location of this disease is the liver and that the failure of sentiment leads to liver Qi stagnation, endogenous heat deficiency, and Yin injury [21]. The fourth view is the theory of spleen and stomach. According to the symptoms that *Golden Chamber* described, TCM experts considered that its pathogenesis lies in the damage of coke due to irregular diet [22].

In summary, the cause of lily disease is the damage of viscera by external or internal injury, leading to Yin deficiency of viscera and disorder of Qi and blood; then, the veins will lose nourishment, and hence, it will lead to the symptoms of mental disorder. The Yin deficiency of viscera will lead to the virtual heat coming from the inside of the body, as well as bitterness in the mouth, red-colored urine, and other symptoms.

## 3. Yin Deficiency and Internal Heat of Depression

According to the TCM theory, depression can be divided into positive syndromes and deficiency syndromes, including Qi stagnation, fire stagnation, phlegm stagnation, blood stagnation, heart and spirit loss nourish, deficiency of both heart and spleen, and hyperactivity of fire due to Yin deficiency. Most of the Yin deficiency internal heat depression is menopausal depression or depression in old age, and its etiology is plant nerve dysfunction and endocrine dysfunction [23].

Contemporary scholars believe that depression belongs to the category of “no disease” in TCM. Most patients have no obvious organic lesions. The pathogenesis is the imbalance of Qi, blood, Yin, Yang, and the dysfunction of viscera, which makes the body in a state of excessive Yin and Yang or partial decline. Disorders of Qi and blood in the viscera will lead to abnormal mind, and changes in mind will also affect Qi and blood in the five viscera [24].

TCM experts generally considered that depression is closely related to Qi and blood blockage and liver unreel distributing [25, 26]. All kinds of negative emotional stimulation, such as mental panic, physical and mental exhaustion, and failure to achieve one's goals, can cause dysfunction of viscera, Qi, and blood and lead to depression. This disease was sufficiency syndrome at first and can be triggered by the internal heat due to Qi stagnation, then pathogenic factors will enter the blood from Qi, and the disease changed from sufficiency syndrome to deficiency syndrome, so there will be a mixture of deficiency and excess syndromes such as the deficiency of mind, deficiency of Qi and Yin, Yin deficiency, and fire excess [27].

The intrinsic mechanism of Yin deficiency and internal heat of depression is the imbalance of the kidney, lung, and spleen and other viscera. The clinical manifestation of Yin deficiency and internal heat of depression is a mixture of deficiency and excess syndromes [28]. Vital Qi deficiency is due to the lack of Yin and fluid, and after a while, it will become Qi and Yin deficiency. Thus, pathogenic factor excess is because of the body heat and blood stasis. The syndrome of Yin deficiency and internal heat of depression is deficiency in origin and excess in superficiality [29]. It consumes Yin and fluid at first, then feeling the pathogenic factor outside or modern internal injuries, and producing internal heat. The heat transmission will increase the loss of body fluid, then the Yin will not gather the Yang, and the empty fire is burning more. The lack of dirty Yin and spirit will lead to anxiety, depression, tension, and suspicion, and the internal heat due to Yin deficiency disturbance heart will lead to palpitations and insomnia [30].

## 4. Association of Yin Deficiency and Internal Heat of Depression and Lily Disease

Based on the TCM theory, lily disease is a kind of emotional disease, which is closely related to emotional and psychological factors. Lily disease occurs because of the disorder of seven emotions, which will hurt viscera and lead to the imbalance of Qi, blood, Yin, and Yang. In addition, it may be the sequela of exogenous fever or other diseases [31]. With regard to etiology, the pathogenesis of lily disease due to modern factors was similar to that of depression due to personality or negative emotional experience. In addition to emotional factors, lily disease is also related to exogenous febrile disease.

From the pathogenesis, the basic pathogenesis of lily disease is internal heat due to Yin deficiency, and its pathogenesis is the result of the interaction of various pathogenic factors [32]. From the clinical symptoms, lily disease showed trance, sleep, anxiety, insomnia, and other symptoms similar to the clinical diagnostic standard of modern depression. Besides, bitterness in the mouth, red-colored urine, and rapid and thready pulse are the typical symptoms of internal heat due to Yin deficiency [33]. Therefore, we can infer that internal heat depression due to Yin deficiency is major syndrome of lily disease from etiology and pathogenesis.

## 5. Traditional Chinese Formulas for Treatment of Lily Disease

Zhang Zhongjing believed that the treatment of emotional diseases should first improve the physical symptoms combined with Chinese herb treatment based on syndrome differentiation [34]. The main prescription for treating lily disease is Lily Bulb and Rehmannia Decoction (LBRD). The classical herbal formula LBRD is combined with the lily bulb and fresh Romanian root juice. According to TCM perspective, lily bulb is deemed cool and sweet in properties. The lily bulb is also associated to the lung and heart meridians and help to relieve cough and dry throat, clear heat and moisten the lung. *Rehmannia* root is naturally sweet with bitter flavor, and mostly shows its curative effects in the kidney, liver, and heart. It has effect on promoting body fluid production, nourishing Yin, and controlling heat [11]. When two ingredients are combined, LBRD will bring about the maximum of therapeutic efficacy on the mental instability, anhedonia, anxiety, absent mindedness, and insomnia.

Lily Zhimu Decoction (BZD) was another traditional Chinese formula for treatment of lily disease [35]. Zhimu (*Anemarrhena*) is bitter and sweet in properties, and it is also associated to the lung, stomach, and kidney meridian. It has the effect of clearing away heat, eliminating annoyance, draining the lungs, and nourishing the kidneys. Because “blood and sweat are homologous” and “sweat and fluid are homologous,” excessive sweating will lead to more deficiency of body fluid, Qi and blood, and obvious symptoms of dry thirst [36].

According to the TCM theory, Talc Hematite Decoction (THD) was used to treat lily disease mistreated with purgative. Talc tastes sweet and is cold in nature. It was used to treat vexation, hot, and thirsty. Hematite tastes bitter and is flat in nature, into Stomach Channel of Foot-Yang Ming. It can reduce stomach gas to stop hiccups, relieve cough, and clear heat and have the characteristic of inducing astringency without hurting vital energy. After the wrong purgation, stomach Qi and body fluid were injured, and internal heat increased. Because the main disease has not changed, lily is still the monarch medicine. Talc is used as minister medicine to diuretic and purgation heat, and hematite is used as adjuvant drug to induce astringency and astringing Yin [37].

Lily disease mistreated with the vomiting method should be treated with Lily Yolk Decoction (LYD) underlying syndrome differentiation [21]. Yolk tastes sweat and is warm in nature, it can nourish the spleen and stomach and adjust heart gas to nourish Yin, and it has the effect of clearing heat to cool blood and detoxification. The erroneously treatment of the vomiting method will injure stomach Yin and disturbs lung and stomach Qi, leading to restlessness due to deficiency.

Lily disease patients with lasting for one month without alleviation can use lily to wash the body. If thirst is severe, it should be treated with Gualou oyster powder (GOP) [38]. Trichosanthin tastes bitter and is cold in nature, mainly used to treat thirst, except for body heat and irritation, and it can nourish body fluid without damaging the Yang of the spleen and stomach; oyster tastes salty and is acerbity, cold in nature, and heavy in quality, which has the effect of reducing virtual heat and avoid draining the body fluid. Both of these two medicines can remove heat and supplement Yin-Jin to relieve thirst. Lily disease with thirst by GOP and the oyster in the formula did not act directly to regenerate body fluid and quench thirst, but to check exuberance of Yang, so that the Yin would rest the Yang, and the thirst would be quenched.

## 6. Traditional Chinese Herb Prescription for Treatment of Yin Deficiency and Internal Heat of Depression

**Figure 2:**
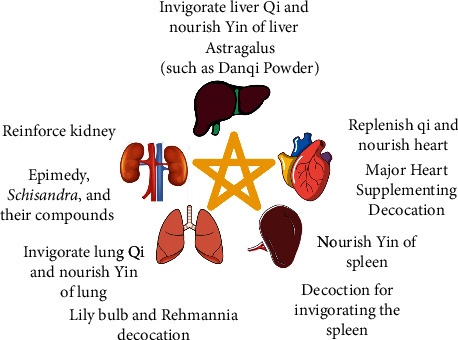
Traditional Chinese medicine for treatment of Yin deficiency and internal heat of depression.

TCM believes that the pathogenesis of depression is related to the deficiency of Qi and Yin in several viscera. Therefore, the main principles for treating Yin deficiency and internal heat of depression are nourishing Yin to clear heat in five viscera (Figure 2). However, the pathogenesis of Yin deficiency and internal heat of depression is complex and the viscera interact with each other. Thus, when treating from one viscus, other viscera should be considered at the same time.

Heart Qi deficiency can lead to poor blood flow and blockage of heart vessel. According to *the Canon of Internal Medicine*, people's spiritual consciousness and thinking activities are coordinated by the five viscera. The heart is the master of all the viscera recorded in *Lingjiu*. It means that the heart has a controlling effect on the five viscera, and the malfunction of the heart can also lead to the malfunction of other viscera. The treatment of Yin deficiency and internal heat of depression from the heart mainly adopts the method of replenishing Qi and nourishing the heart. Major Heart Supplementing Decoction was clinically used to treat heart Qi deficiency-type depression [31]. Cortex Moutan acts on blood, *Inula japonica* acts on Qi, and the two drugs used mutual promotion can benefit heart Qi and enhance heart function. Bamboo leaves can clear the mind and purge fire to remove irritation. Pulp of *Cornus* can ease the emergency of the heart and prevent dredge too much, leading to the consumption of Qi. Ginseng and dried ginger can benefit heart Qi to enhance heart function. This prescription has the effect of supplementing heart Qi. It is an effective prescription for treating depression due to heart Qi deficiency.

Yin deficiency and hot of the lung can lead to the failure of the lung to disperse and descend. The treatment of Yin deficiency and internal heat of depression from the lung is mainly based on the method of invigorating Qi and nourishing Yin. Zhang et al. considered that LBRD has a good therapeutic effect in clinical practice [15]. In this prescription, lily can moisten the lung to benefit Qi and clear heat to calm the mind. *Rehmannia* can nourish the heart and Yin, as well as cool blood to clear heat, and the two drugs can nourish the heart and lung and clear heat to calm nerves when they are used together.

Lack of body fluid in the spleen makes it become hot and dry and leads to Yin deficiency, which will cause the maladjustment of transport and transformation of the spleen. The spleen is the most negative viscera in the human body. And spleen Yin deficiency will affect the lung, liver, and kidney functions and result in the dry heat performance of the body [39]. Therefore, the treatment of Yin deficiency and internal heat of depression from the spleen mainly adopts the method of nourishing spleen Yin. For example, professor Liu found that decoction for invigorating the spleen has a good therapeutic effect on depression through experiments [40]. In this prescription, *Angelica sinensis*, *Astragalus*, and Arillus Longan have the effect of invigorating Qi, activating blood, and calming spirits. Lanceolata can nourish Qi and enhance the function of the spleen and lungs. *Atractylodes*, *Poria cocos*, and spine date seed have the function of calming the mind and eliminating dampness to strengthening the spleen. This prescription has the function of strengthening the spleen and blood to calm the spirit, and it is an effective prescription for the treatment of depression due to spleen deficiency.

Liver Qi stagnation will make the liver hot and then damage the liver Yin. If the dredge function of the liver is abnormal, the operation of Qi in the viscera and meridians will be obstructed, which will lead to the damage of liver Yin and the appearance of internal heat. The treatment of Yin deficiency and internal heat of depression from the liver is mainly based on the methods of invigorating liver Qi and nourishing Yin of the liver. Common formulations include Ease Powder, Bupleurum Decoction, and so on [41]. In addition, *Astragalus* is commonly used in clinical practice to replenish liver Qi. For example, Wang et al. [21] treat depression due to liver Qi deficiency with self-designed empirical formula “Danqi Powder.” *Astragalus* can replenish Qi. Salvia can activate and nourish blood. *Pinellia ternata* tastes spicy and can resuscitate and dissipate the lump, and it is an adjuvant drug to inhibit the disadvantage of a large amount of *Astragalus*. The combination of these three drugs can invigorate Qi and activate blood circulation and has the advantage of supplementing without stagnation.

The kidney essence exhausted will make the kidney dry and hot and lead to the injury of kidney Yin. The kidney is the origin of congenital constitution and holds essence, and the essence of the kidney is the material basis of kidney Yin. It shows that the reason of kidney Yin deficiency is the insufficieny of kidney essence. The treatment of Yin deficiency and internal heat depression from the kidney is mainly based on the method of reinforcing the kidney [42]. Epicedium can warm the kidney to strengthen Yang and remove depression to anchore mind [43]. *Schisandra chinensis* has many functions, including tonifying the lung to collect Qi, nourishing the heart to remove annoyance and tranquility, strengthening the spleen and consolidating the base, regulating the rise and fall, softening the liver to relieve depression, and invigorating Qi to stabilize kidney essence [44]. These two drugs can be used according to the symptoms of depression caused by the essence insufficiency of the kidney.

## 7. Coronavirus Disease 2019 and Yin Deficiency and Internal Heat of Depression

Coronavirus disease 2019 (COVID-19) has the characteristics of fast transmission, wide transmission, strong infection, general population susceptibility, and no specific therapeutic agents [45]. It is an emergent public health security event, and it not only causes physical damage to the body but also stimulates the psychological balance of the public. Recent research shows that anxiety is the highest incidence of psychological problems among the public during the epidemic, followed by depression and insomnia [46, 47].

TCM experts believe that COVID-19 was classified as “epidemic disease,” with the lung and spleen as the main disease sites [48, 49]. Qi, blood, and body fluid syndrome differentiation can be divided into Qi deficiency, Qi and blood deficiency, Qi inverse, and body fluid loss; the differentiation of viscera is related to the five viscera. It can be seen that novel coronavirus pneumonia and lily disease are all located in the lung, which is closely related to the deficiency of Qi and Yin in the lung and dysfunction of the viscera [50]. Therefore, we guess COVID-19 may cause lily disease. In addition, lily disease belongs to “depression syndrome,” which is closely related to emotion. TCM believes that grief damages the spleen, and it is also one of the main diseases of COVIN-19. When the novel coronavirus pneumonia causes lily disease in the later stage, it may aggravate the disease and is not conducive to the treatment of the disease.

The pathogenesis of COVID-19 varies from mild to severe, from surface to entrant, and from solid to deficiency [51, 52]. The pathogenesis is characterized by heat and located in the lung. At the beginning of the disease, the stomach accepts the pathogenic factor, so there are nasal congestion, runny nose, sore throat, and other symptoms. Pathogenic virus infected the lung and decreased its dysfunction of controlling dispersing outwards and inwards. Pathogenic virus depleted vital Qi and caused muscles to become tired and sore. Wind chill bounded body surface, which leads to tongue coating with thin white or slightly greasy and pulse with floating tight. In the middle of the disease, pathogens block the lung, and depression causes internal heat and consumes body fluid, so the symptoms of high fever and thirst appear. Phlegm is obstructed, and Qi is stagnant, so bosom frowsty wheeze and breath are difficult [53]. Hot phlegm fills the lungs, so phlegm is sticky and mood is agitated, and at the same time, it is accompanied by short urine and constipation symptoms. In this period, some patients also have a series of typical Shaoyang syndrome such as depressed, upset, chest tightness, fever, bitterness in the mouth , poor appetite, nausea, and vomiting. In the later stage of the disease, many patients show cough, little sputum, mental exhaustion, lack of speech, thirst, poor absorption, dark red tongue with less moss, and thready or weak pulse. This is because in the later stage of the disease, the residual heat is not solved, and the spleen and stomach are damaged [54].

A recent study indicates that exogenous febrile disease and pulmonary diseases are related to the occurrence of lily disease [55]. From the perspective of TCM, COVID-19 patients are prone to lily disease in the middle and later stages of the disease. It is because that lung damage causes the malfunction of spreading and declining, and deficiency of Qi and Yin in the lungs will lead to hot and dry. If the residual heat in the late period of febrile disease has not been solved, it will consume Yin-Jin and lead to lily disease. From the perspective of sociology, COVID-19 patients often have feelings of longing for family ties, fearing of disease and the stigma of infection, loneliness, and guilt. Then, it will cause symptoms of anxiety and depression. Emotional failure will damage the Qi and blood functions of viscera and lead to the occurrence of lily disease.

TCM has been widely recognized and widely applied in the treatment of COVID-19 during the epidemic. COVID-19 prevention and treatment program and clinical prescription data in various regions of China suggested that the common high-frequency drugs of the prevention and treatment plan and clinical medical records included *Glycyrrhiza*, *Pogostemon cablin*, bitter almond, honeysuckle, *Poria cocos*, weeping forsythia, tangerine peel, and *Coix* seed [56]. Currently, Chinese patent medicine for treatment of COVID-19 is mainly included Jinhua Qinggan granule, Lianhuaqingwen capsule, etc. In addition, some researchers believe that moxibustion [57], fragrant medicines, and scraping can be applied to the prevention and treatment of COVID-19.

We believe that the treatment prescription of lily disease can be considered as a supplementary treatment for pneumonia in the middle and later stages, to clearing away heat and nourishing Yin, strengthening spleen and benefiting vital Qi, and regulating the function of viscera, Qi, and blood, which is beneficial for novel coronavirus pneumonia rehabilitation.

COVID-19 patient medical therapy should be paid not only to the treatment of pneumonia itself but also to the psychological state of patients. The patients' mood changes will affect the treatment and prognosis of the disease. So, it is necessary to carry out psychological intervention and drug therapy for emotional diseases. In addition, COVID-19 patients will still have different psychological problems after recovery and discharge from hospital, and follow-up and psychological intervention after discharge is still an urgent task.

## 8. Conclusion and Perspective

We systematically summarize the research status of lily disease and Yin deficiency and internal heat of depression and came to the conclusion that the cause of lily disease is the damage of viscera by external or internal injury, which will lead to Yin deficiency of viscera, and the disorder of Qi and blood; then, the veins will lose nourishment, and hence, it will lead to the symptoms of mental disorder. The Yin deficiency of viscera will lead to the virtual heat coming from the inside of the body, as well as bitterness in the mouth, red-colored urine, and other symptoms.

From the clinical symptoms, lily disease showed trance, sleep, anxiety, insomnia, and other symptoms similar to the clinical diagnostic standard of modern depression. And bitterness in the mouth, red-colored urine, and rapid and thready pulse are the typical symptoms of internal heat due to Yin deficiency. Therefore, we can infer that internal heat depression due to Yin deficiency is a major syndrome of lily disease from etiology and pathogenesis.

For COVID-19 patients, lung damage can cause spread and decline malfunction, and deficiency of Qi and Yin in the lungs will make it hot and dry. If the residual heat in the late period of febrile disease has not been solved, it will consume Yin-Jin and result in lily disease. Therefore, the treatment of lily disease can also be referenced when treating COVID-19 patients.

## Data Availability

The data used to support the findings of this study are available from the corresponding author upon request.
